# Two-Year Toxicity and Efficacy of Carbon Ion Radiotherapy in the Treatment of Localized Prostate Cancer: A Single-Centered Study

**DOI:** 10.3389/fonc.2021.808216

**Published:** 2022-02-11

**Authors:** Ping Li, Zhengshan Hong, Yongqiang Li, Shen Fu, Qing Zhang

**Affiliations:** ^1^ Department of Radiation Oncology, Shanghai Proton and Heavy Ion Center, Shanghai, China; ^2^ Shanghai Engineering Research Center of Proton and Heavy Ion Radiation Therapy, Shanghai, China; ^3^ Shanghai Key Laboratory of Radiation Oncology, Shanghai, China; ^4^ Department of Medical Physics, Shanghai Proton and Heavy Ion Center, Shanghai, China; ^5^ Key Laboratory of Nuclear Physics and Ion-Beam Application (MOE), Fudan University, Shanghai, China; ^6^ Department of Radiation Oncology, Shanghai Concord Cancer Hospital, Shanghai, China

**Keywords:** prostate cancer, carbon ion radiotherapy, spot scanning, local effect model, toxicity

## Abstract

**Background:**

We aimed at determining the safety and feasibility of spot-scanning carbon ion radiotherapy (CIRT) for patients with localized prostate cancer.

**Methods:**

We enrolled 118 patients with localized prostate cancer who underwent treatment with spot-scanning CIRT at the Shanghai Proton and Heavy Ion Center (SPHIC) from January 2016 to December 2020. The dose was gradually increased from relative biological effectiveness (RBE)-weighted dose (D_RBE_) = 59.2–65.6 Gy in 16 fractions. The primary endpoint was the occurrence of acute and late toxicities, while the secondary endpoints were biochemical relapse-free survival (bRFS), distant metastasis-free survival (DMFS), prostate cancer-specific survival (PCSS), and overall survival (OS).

**Results:**

The median follow-up time was 30.2 months (4.8–62.7 months). Acute grade 1 and 2 genitourinary (GU) toxicities were 15.3% and 18.6%, while acute grade 1 and 2 gastrointestinal (GI) toxicities were 2.5% and 0%, respectively. Late grade 1 and 2 GU toxicities were 4.2% and 1.7%, respectively. No late GI toxicity was observed. Moreover, there were no cases of severe acute or late toxicity (≥ grade 3). No significant association were observed between the factors and the acute GU toxicities, except for clinical target volume (CTV) (p = 0.031) on multivariate analysis. The 2-year bRFS, DMFS, PCSS, and OS were 100%, 100%, 100%, and 98.8%, respectively.

**Conclusion:**

The 2-year outcomes were encouraging, providing additional and useful information on the feasibility and safety of spot-scanning CIRT for treating prostate cancer. Thus, we recommend long-term follow-up and prospective multicentered studies to reinforce the role of CIRT in the management of localized prostate cancer.

## Introduction

Radiotherapy is a radical treatment option for localized prostate cancer. Randomized studies have demonstrated that dose-escalated radiotherapy improves cancer control (1, 2). However, increasing the dose leads to concerns about the toxicities in organs at risks (OARs), such as the rectum and bladder. Thus, if the dose to the prostate can be increased without increasing the dose to the OARs, the treatment outcome and quality of life of patients will be improved.

Carbon ion radiotherapy (CIRT) can minimize radiation dose to OARs while increasing the biologically effective dose delivery to the prostate (3, 4). According to the data from the Particle Therapy Co-operative Group (https://www.ptcog.ch/) on June 2021, approximately 40,000 patients have been treated with CIRT in 12 carbon ion centers worldwide. The higher relative biological effectiveness (RBE) and greater cytocidal effect of CIRT on cancer cells make it more beneficial over conventional radiotherapy (5). Additionally, carbon ion beams produce the Bragg peak through the release of enormous energy at the end of their range (6), maximizing the destructive energy delivered to the tumor site while minimizing unwanted damage to the surrounding normal tissues (7, 8). These properties make CIRT theoretically efficient in improving tumor control and reducing radiation-related toxicity.

The first CIRT clinical trial for prostate cancer was started in Chiba, Japan in 1995 (8). This phase I/II dose escalation study established the efficacy and safety of CIRT. The following studies in Japan further confirmed the effectiveness of the CIRT with 16-fraction regimens (4, 9). However, different models were used to predict the RBE at different institutes. Two major RBE models have been applied clinically in CIRT: the Japanese model [the original mixed beam model and the modified micro-dosimetric kinetic model (MKM)] and Helmholtzzentrum für Schwerionenforschung GmbH (GSI) model [local effect model (LEM)] (10, 11). Comparative studies showed that the LEM predicts a 5%–15% higher RBE in the spread-out Bragg peak of a carbon ion beam, relative to the MKM (12). Similarly, findings from previous studies revealed that the RBE-weighted doses using MKM for targets and OARs should be converted to LEM doses using conversion curves for prostate cancer treated with CIRT (13). This hampers the exchange of experience between different CIRT facilities by the use of disparate RBE models. Thus, assessing the dose needed when using LEM model to achieve results similar to those reported by Japanese facilities will be of great value.

In 2014, the Shanghai Proton and Heavy Ion Center (SPHIC) started the first prostate cancer CIRT treatment in China. Till January 2021, more than 300 patients with prostate cancer have undergone particle therapy at our institute, including patients with localized prostate cancer, oligo-metastatic prostate cancer, prostate cancer with pelvic lymph node metastasis, and postoperative prostate cancer. To establish the optimal dose for CIRT in LEM model, dose-escalated clinical trials of CIRT for patients with localized prostate cancer began in January 2016 at our center (NCT02739659 and NCT04724577). The purpose of this study was to assess the 2-year toxicity, biochemical relapse-free survival (bRFS), distant metastasis-free survival (DMFS), prostate cancer-specific survival (PCSS), and overall survival (OS) of the 118 patients treated with CIRT in 16 fractions.

## Patients and Methods

### Study Design and Patient Eligibility

We consecutively enrolled 118 patients with localized prostate cancer treated with 16 fractions CIRT at SPHIC between January 2016 and December 2020 through a retrospective design. Patients were included if they meet the following: (1) histological diagnosis of prostate adenocarcinoma, (2) cT1N0M0 to cT4N0M0 according to the 7th American Joint Committee on Cancer (AJCC) classification, (3) Karnofsky Performance Score ≥70, (4) without any previous surgery or radiotherapy for prostate cancer, and (5) the presence of written informed consent. We excluded patients who did not meet all of the aforementioned criteria.

The study was approved by the Institutional Review Board of SPHIC (Approval Number 180620EXP-02). Eligible patients gave their written informed consent for CIRT and for future anonymous use. All patients were treated by spot-scanning CIRT combined with or without hormone therapy in our institute. According to the National Comprehensive Cancer Network (NCCN) guidelines, patients with low-risk prostate cancer had no hormone therapy, while intermediate-risk patients received 4–6 months of hormone therapy, and high/very high-risk patients received hormone therapy for 2–3 years. The hormone therapy regimens were combined androgen blockade.

### Carbon Ion Radiotherapy

Methods for preparing the bladder and rectum, immobilizing the patients, and setting clinical target volume (CTV) and planning target volume (PTV) have been described (14). Briefly, the CTV routinely included the prostate and seminal vesicle (seminal vesicle was excluded for low-risk patients), and pelvic lymph nodes were excluded from this study. Two opposite lateral beams were used for each fraction treatment. CIRT was given once a day, five fractions per week. The treatment position was adjusted before each fraction with orthogonal X-ray scans as image guide. Since May 2020, the daily in-room computed tomography (CT) was applied to guide the CIRT. The prescription was performed in terms of RBE-weighted dose (D_RBE_). RBE was calculated by the treatment planning system (Syngo), using the LEM model. The carbon ion was administered at a five-dose regimen of D_RBE_ = 59.2/60.8/62.4/64/65.6 Gy in 16 fractions. OARs required for all the patients were the rectum and the bladder. The dose constraints of the rectum are as follow: Dmax (the max dose) <105% prescription dose (PD), V_60_ [volume receiving ≥ 60 Gy < 3 cc], V_55_ [volume receiving ≥ 55 Gy < 7 cc, and V_50_ [volume receiving ≥ 50 Gy] < 10 cc, which were referred from our previous dose conversion study (13, 14). The dose constraints on the bladder were Dmax <105% PD and V_60_ [volume receiving ≥ 60 Gy] < 10%, V_55_ [volume receiving ≥ 55 Gy] < 15%, and V_30_ [volume receiving ≥ 30 Gy] < 30%.

### Follow-Up

To closely monitor the patients, the patients’ follow-up was performed every week during treatment, and every 3 months until 3 years after CIRT, then sixth monthly until further notice. A rise in prostate-specific antigen (PSA) by at least 2 ng/ml above the nadir (the Phoenix definition) is considered as biochemical failure (15). For each patient, baseline parameters for genitourinary (GU) and gastrointestinal (GI) functions were assessed and acute, and late toxicities were scored by a physician using Common Terminology Criteria for Adverse Events v.4.03 and Radiation Therapy Oncology Group (RTOG) Classification (16). Acute toxicities are defined as side effects occurring within 3 months after the start of CIRT. Toxicities that occurred 3 months after the start of CIRT were considered late toxicities.

### Statistics

The bRFS, DMFS, PCSS, and OS were evaluated using the Kaplan–Meier method. The bRFS, DMFS, PCSS, and OS were calculated from the start date of CIRT. The chi-square test was used to examine the difference in acute GU toxicities between the low-dose [D_RBE_ = 59.2–60.8 Gy] and high-dose [D_RBE_ = 62.4–65.6 Gy] groups. Logistic and Cox regression identified univariate and multivariate associations between toxicities and clinical/dosimetric characteristics. A two-sided p < 0.05 was considered statistically significant. All the analyses were performed using SPSS software (version 22.0; IBM Corp.).

## Results

### Patients Characteristics

**Table 1** shows the characteristics of the included patients. Their median age of the patients was 71 years old (range, 46–86 years). The median follow-up time was 30.2 months (4.8–62.7 months). According to the 7th AJCC classification, the number of patients with T1, T2, T3, and T4 was 5 (4.2%), 94 (79.7%), 17 (14.4%), and 2 (1.7%), respectively. The number of patients with Gleason score of 6, 7, and ≥8 was 32 (27.1%), 46 (39.0%), and 40 (33.9%), respectively. Before treatment, 45 patients had a PSA level <10 ng/ml, 42 had a PSA level ranging from 10 to 20 ng/ml, and 31 had a PSA level >20 ng/ml. According to the NCCN guideline, the number of patients at low-risk, intermediate-risk, and high/very-high risk patients was 9 (7.6%), 45 (38.1%), and 64 (54.2%), respectively. The number of patients irradiated with an RBE-weighted dose of 59.2, 60.8, 62.4, 64.0, and 65.6 Gy was 43 (36.4%), 10 (8.5%), 9 (7.6%), 25 (21.2%), and 31 (26.3%), respectively. All patients completed their spot-scanning CIRT.

**Table 1:** Patients’ characteristics.

Characteristics	D_RBE_ = 59.2 Gy	D_RBE_ = 60.8 Gy	D_RBE_ = 62.4 Gy	D_RBE_ = 64.0 Gy	D_RBE_ = 65.6 Gy	Total
**Patient number**	43 (36.4%)	10 (8.5%)	9 (7.6%)	25 (21.2%)	31 (26.3%)	118(100%)
**BED (α/β = 1.5 Gy)**	205.2 Gy	214.8 Gy	224.6 Gy	237.4 Gy	244.9 Gy	NA
**Age (year)**						
Median (range)	69 (50–84)	73 (69–79)	68 (62–74)	72 (47–86)	73 (50–86)	71 (46–86)
**T stage**						
T1	4 (9.3%)	0 (0%)	0 (0%)	0 (0%)	1 (3.2%)	5 (4.2%)
T2	32 (74.4%)	8 (80.0%)	8 (88.9%)	22 (88.0%)	24 (77.4%)	94 (79.7%)
T3	5 (11.6%)	2 (20.0%)	1 (11.1%)	3 (12.0%)	6 (19.4%)	17 (14.4%)
T4	2 (4.7%)	0 (0%)	0 (0%)	0 (0%)	0 (0%)	2 (1.7%)
**Initial PSA (ng/ml)**						
<10	13 (30.2%)	5 (50.0%)	4 (44.4%)	10 (40.0%)	13 (41.9%)	45 (38.1%)
≥10 and ≤20	16 (37.2%)	2 (20.0%)	2 (22.2%)	11 (44.0%)	11 (35.5%)	42 (35.6%)
>20	14 (32.6%)	3 (30.0%)	3 (33.3%)	4 (16.0%)	7 (22.6%)	31 (26.3%)
**Gleason Score**						
6	13 (30.2%)	3 (30.0%)	2 (22.2%)	4 (16.0%)	10 (29.1%)	32 (27.1%)
7	19 (44.2%)	3 (30.0%)	4 (44.4%)	8 (32.0%)	12 (40.0%)	46 (39.0%)
≥8	11 (25.6%)	4 (40.0%)	3 (33.3%)	13 (52.0%)	9 (30.9%)	40 (33.9%)
**Risk (NCCN)**						
Low	4 (9.3%)	0 (0%)	1 (11.1%)	1 (4.0%)	3 (9.7%)	9 (7.6%)
Intermediate	14 (32.6%)	4 (40.0%)	3 (33.3%)	10 (40.0%)	14 (45.2%)	45 (38.1%)
High/Very High	25 (58.1%)	6 (60.0%)	5 (55.6%)	14 (56.0%)	14 (45.2%)	64 (54.2%)
**Complications**						
Diabetes mellitus	7 (16.3%)	0 (0%)	1 (11.1%)	7 (28.0%)	4 (12.9%)	19 (16.1%)
Internal use of anticoagulanti	6 (14.0%)	0 (0%)	0 (0%)	3 (12.0%)	4 (12.9%)	13 (11.0%)
TURP	4 (9.3%)	1 (10%)	0 (0%)	2 (8.0%)	3 (9.7%)	10 (8.5%)

BED, biological equivalent dose; RBE, relative biological effectiveness; D_RBE_, RBE weighted dose; PSA, prostate-specific antigen; NCCN, National Comprehensive Cancer Network; TURP, transurethral resection of the prostate; Na, not applicable.

### Acute Toxicities

All patients were included in the analysis of acute and late toxicities. The acute toxicities are summarized in **Table 2**. The incidences of grades 1 and 2 acute GU toxicities were 15.3% and 18.6%, respectively. Moreover, eight (15.1%) and five (9.4%) patients developed grade 1 and 2 acute GU toxicities in the low-dose group, respectively, while 10 (15.4%) and 17 (26.2%) patients developed grade 1 and 2 acute GU toxicities in the high-dose group, respectively. The incidence of grade 2 acute GU toxicities of the high-dose group was higher than the low-dose group, although not significantly (p = 0.059). There were no significant differences in the frequency of acute GU toxicities, including hematuria, urinary frequency, urgency, retention, and urinary tract pain between the two groups (**Table 2**). CTV volume, bladder V60, V61, V62, and V63 were associated with ≥ grade 1 acute GU toxicities on univariate analysis, but only CTV volume was associated with ≥ grade 1 acute GU toxicities on multivariate analysis (**Table 3**).

**Table 2:** Acute toxicity between low-dose and high-dose groups.

Total dose	D_RBE_ = 59.2–60.8 Gy (n = 53)				D_RBE_ = 62.4–65.6 Gy (n = 65)				*P* value
Toxicity (grade)	0	1	2	≥ 3	0	1	2	≥ 3	
GU									
Max toxicity	40 (75.5%)	8 (15.1%)	5 (9.4%)	0 (0%)	38 (58.5%)	10 (15.4%)	17 (26.2%)	0 (0%)	0.059
Urinary frequency	43 (81.1%)	5 (9.4%)	5 (9.4%)	0 (0%)	41 (63.1%)	9 (13.8%)	15 (23.1%)	0 (0%)	0.081
Urinary urgency	47 (88.7%)	3 (5.7%)	3 (5.7%)	0 (0%)	57 (87.7%)	2 (3.1%)	6 (9.2%)	0 (0%)	0.622
Urinary tract pain	52 (98.1%)	1 (1.9%)	0 (0%)	0 (0%)	62 (95.4%)	1 (1.5%)	2 (3.1%)	0 (0%)	0.433
Hematuria	51 (96.2%)	2 (3.8%)	0 (0%)	0 (0%)	61 (93.8%)	3 (4.6%)	1 (1.5%)	0 (0%)	0.644
Urinary retention	52 (98.1%)	0 (0%)	1 (1.9%)	0 (0%)	60 (92.3%)	3 (4.6%)	2 (3.1%)	0 (0%)	0.258
GI									
Max toxicity	52 (98.1%)	1 (1.9%)	0 (0%)	0 (0%)	63 (96.4%)	2 (3.6%)	0 (0%)	0 (0%)	0.683
Hematochezia	52 (98.1%)	1 (1.9%)	0 (0%)	0 (0%)	64 (98.2%)	1 (1.8%)	0 (0%)	0 (0%)	0.884
Diarrhea	53 (100%)	0 (0%)	0 (0%)	0 (0%)	64 (98.2%)	1 (1.8%)	0 (0%)	0 (0%)	0.364

GU, genitourinary; GI, gastrointestinal.

**Table 3:** Univariate and multivariate clinical and DVH associations with acute GU toxicities (≥ grade 1).

Variable	Univariate Cox regression		Multivariate Cox regression	
	OR (95% CI)	*p*-value	OR (95% CI)	*p*-value
Age	1.001 (0.956–1.047)	0.976	1.003 (0.952–1.056)	0.925
Diabetes mellitus	1.531 (0.509–4.605)	0.448	3.703 (0.881–15.564)	0.074
Internal use of anticoagulant	0.559 (0.174–1.790)	0.327	0.370 (0.077–1.775)	0.214
TURP	2.171 (0.439–10.745)	0.342	1.871 (0.275–12.730)	0.522
CTV volume	1.016 (1.004–1.029)	0.011	1.016 (1.001–1.031)	0.031
Bladder volume	1.005 (0.997–1.013)	0.212	1.002 (0.992–1.013)	0.673
Bladder Dmax	1.105 (0.985–1.240)	0.090	1.038 (0.919–1.173)	0.549
Bladder V30	1.022 (0.983–1.063)	0.275	0.878 (0.463–1.664)	0.690
Bladder V40	1.030 (0.982–1.080)	0.224	1.031 (0.319–3.336)	0.959
Bladder V50	1.045 (0.982–1.111)	0.164	1.700 (0.489–5.912)	0.404
Bladder V55	1.059 (0.985–1.138)	0.122	0.503 (0.167–1.513)	0.221
Bladder V60	1.092 (1.004–1.187)	0.040	1.311 (0.443–3.881)	0.624
Bladder V61	1.106 (1.006–1.215)	0.038	1.193 (0.147–9.673)	0.869
Bladder V62	1.129 (1.009–1.264)	0.034	0.701 (0.135–3.647)	0.672
Bladder V63	1.178 (1.019–1.362)	0.027	1.432 (0.642–3.192)	0.380
Bladder V65	1.227 (0.908–1.658)	0.182	0.762 (0.442–1.313)	0.328

DVH, dose–volume histograms; GU, genitourinary.

Three (2.5%) patients developed grade 1 acute GI toxicities: one patient in the low-dose group and two patients in the high-dose group (*p* = 0.683). There were no significant differences in the frequency of acute GI toxicities manifested by symptoms, such as hematochezia and diarrhea. No patients demonstrated grade 2 or worse acute GI toxicity.

### Late Toxicities

Grade 1 late GU toxicities did not differ significantly between the low-dose group [2 (3.2%) of 62 patients] and the high-dose group [2 (3.6%) of 56 patients] (*p* = 0.158). Five patients developed grade 1 late GU toxicities; three patients presented with urinary frequency, and two patients presented with microscopic hematuria. Moreover, two patients developed grade 2 late GU toxicity (gross hematuria). No grade ≥3 late GU toxicity was observed across the groups. Furthermore, no patient suffered from late GI toxicity within the follow-up period (**Table 4**).

**Table 4:** Late toxicities between the low- and high-dose groups.

Dose regimens	Number of patients	Number of patients (%) with GU toxicity grade				p-value	Number of patients (%) with GI toxicity grade				p-value
		0	1	2	≥3		0	1	2	≥3	
D_RBE_ = 59.2–60.8Gy	53	52 (98.1%)	1 (1.9%)	0 (0%)	0 (0%)	0.158	53 (100%)	0 (0%)	0 (0%)	0 (0%)	NA
D_RBE_ = 62.4–65.6Gy	65	59 (90.8%)	4 (6.2%)	2 (3.1%)	0 (0%)		65 (100%)	0 (0%)	0 (0%)	0 (0%)	
Total	118	111 (94.1%)	5 (4.2%)	2 (1.7%)	0 (0%)		118 (100%)	0 (0%)	0 (0%)	0 (0%)	

GU, genitourinary; GI, gastrointestinal, NA, not applicable.

### Efficacy

**Figure 1:**
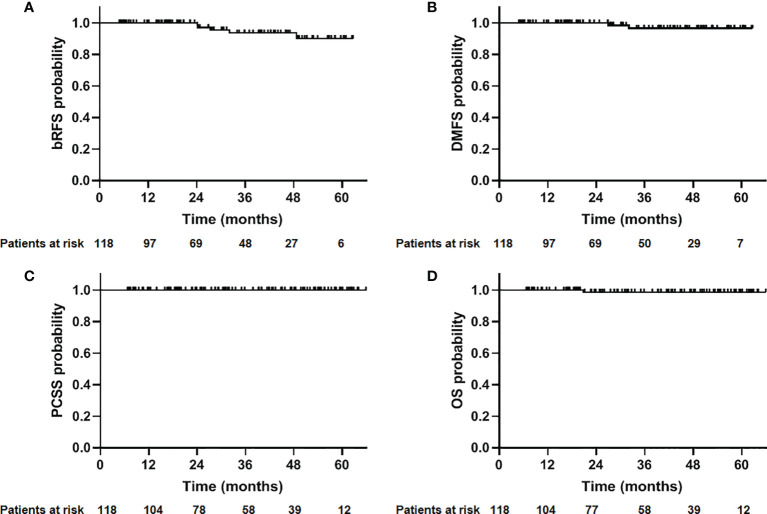
Outcome of 118 patients with prostate cancer treated with CIRT. **(A)** Biochemical relapse-free survival (bRFS); **(B)** distant metastasis free survival (DMFS); **(C)** prostate cancer-specific survival (PCSS); **(D)** overall survival (OS).

**Figure 2:**
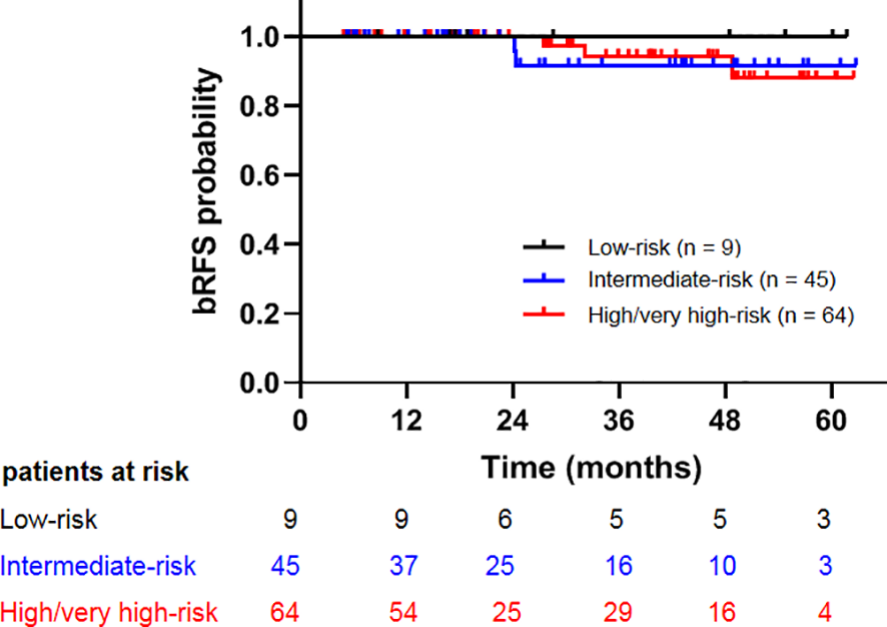
Comparison of biochemical relapse free survival among patients with low-, intermediate-, and high-/very high-risk prostate cancer.

The median follow-up time was 30.2 months (4.8–62.7 months). At the end of follow-up, five patients developed biochemical relapse, two patients received re-biopsy, but there was no evidence of tumor cells, and the PSA were stable without any treatment, such as hormone therapy. One patient received hormone therapy immediately after the diagnosis of PSA failure. Two patients who were classified as high- and very-high risk groups experienced bone metastases at 27.4 and 32.1 months after CIRT, respectively. A 72-year-old patient died at 20.6 months after CIRT due to cerebrovascular accident. No patient died of prostate cancer throughout the observation period. The 2-year bRFS, DMFS, PCSS, and OS was 100%, 100%, 100%, and 98.8%, respectively (**Figure 1**). The 2-year bRFS in low-, intermediate-, and high-/very-high risk groups was 100%, 100%, and 100%, respectively (*p* = 0.782) (**Figure 2**).

## Discussion

The improvement in biochemical relapse-free and OS of patients with prostate cancer was found to be dose dependent (17, 18). Several studies have shown the great potential of CIRT in the management of prostate cancer (3, 4, 8, 9), most from Japanese institutes. However, the biological model used in Europe and our center (LEM model) were different from that used in the Japanese institutes (MKM model) (19). Previous studies reveal that the RBE-weighted doses at our center are too conservative compared with those in Japanese institutes (13). The clinical study also found that the 5-year local control (71%) of skull base chordoma treated with CIRT at Centro Nazionale di Adroterapia Oncologica was inferior to the results reported by Japanese centers (76%–92%) using the same prescription dose, and they found that 92% of the local recurrences were attributable to suboptimal target dose in regions close to the brainstem or optic pathways (12, 20, 21). These studies indicated that the prescription dose from Japanese experiences for prostate cancer could not be replicated. Until now, the optimal dose for CIRT with LEM model in localized prostate cancer is not clear yet.

The first dose escalation clinical trial of CIRT for prostate cancer occurred in Japan. The 5-year bRFS of the 57.6 Gy in a 16-fraction regimen was 88.5% (9). In a multi-institutional study, which collected and re-analyzed data from prospective clinical trials conducted in three institutions in Japan, the 5-year bRFS in low-risk, intermediate-risk, and high-risk patients was 92%, 89%, and 92%, respectively (4). At first, 63/66 Gy in 23/24 fractions was applied to treat patients with prostate cancer in our institute. The dose regimens were well tolerated and without any ≥ grade 2 late GI and GU toxicities. In this study, we retrospectively evaluated the safety and feasibility of CIRT for patients with localized prostate cancer. By this, 118 patients treated by CIRT in our institute achieved satisfactory short-term biochemical control without developing serious adverse events.

In this study, 118 patients were treated with D_RBE_ = 59.2 Gy (n = 43), 60.8 Gy (n = 10), 62.4 Gy (n = 9), 64.0 Gy (n = 25), and 65.6 Gy (n = 31). Five patients developed biochemical relapse, all of which received 59.2Gy group. No patient in the ≥60.8 Gy groups developed biochemical relapse within the follow-up period. The comparative analysis for efficacy between low- and high-dose groups was not performed due to the relatively short follow-up time. The prescribed dose was D_RBE_ = 57.6 Gy in 16 fractions in the National Institute of Radiobiological Science, Japan. Our previous study showed that 3.60 Gy per fraction for 16 fractions in MKM could be converted to 4.21 Gy per fraction for 16 fractions in LEM (13). Hence, further evaluation of the efficacy of the fixed dose at 65.6 Gy in 16 fractions regimen in LEM model system is highly recommended.

In terms of acute toxicity, according to results from the RTOG 0126 clinical trial (22), in 751 patients treated with 79.2 Gy photon therapy, the incidence of grade 1–3 acute GU toxicity was 19%, 16%, and 1%, respectively, and the incidence of grade 1–and 3 acute GI toxicity was 7%, 7%, and <1%, respectively. Moreover, 91 patients were treated with D_RBE_ = 66 Gy in 20 fractions carbon ion or proton in the Ion Prostate Irradiation (IPI) study from Heidelberg Ion-Beam Therapy Center (HIT) (23), and the incidence of grade 1–3 acute GU toxicity was 34.1%, 17.6%, and 0%, respectively, and that of grade 1–3 acute GI toxicity was 60.4%, 7.7%, and 2.2%, respectively. In our study, patients in the high-dose group seem to experience higher rates of acute grade 2 GU toxicities than those in the low-dose group (26.2% vs. 9.4%). No ≥ grade 3 GU toxicity was observed in both groups. The incidence of acute GU toxicities in our study is consistent with that found in 79.2 Gy arms in the RTOG 0126 study but lower than that of the IPI study. The incidence of acute GI toxicity in our study is rare and significantly lower than that of the IPI study. A possible explanation for these differences may lie in that half of the patients received proton therapy in IPI, while daily in-room CT was applied to every patient with prostate cancer in our center since 2020. The association between the prognostic factors and the acute toxicities has been investigated in this study. We found that CTV volume was associated with acute GU toxicities. Previous studies also revealed that patients with large prostate volumes have a great risk of irritative/obstructive symptoms (particularly dysuria) in the acute radiotherapy phase (24). This may be due to the fact that a greater bladder volume was irradiated and lower urinary bother score before CIRT for patient with large prostate was observed. Therefore, for patients with large prostate in our center, neoadjuvant hormone therapy is often recommended to reduce prostate volume.

Late toxicities tend to be more problematic than acute toxicities in radiotherapy for prostate cancer. The incidence rates of late grade 2 and 3 GU toxicities after radiotherapy were 11% and 3%, respectively, in the RTOG 0126 trial (79.2Gy arm) (22). According to the results of a multi-institutional study from the Japan Carbon Ion Radiation Oncology Study Group (J-CROS), which analyzed 2,157 patients treated with CIRT, the incidence rates of late grade 2 and 3 GU toxicities were 4.2% and 0%, respectively (4). In this study, the incidence rates of late grade 2 and 3 GU toxicities were 1.7% and 0%, respectively. The incidence rates of late grade 2 and 3 GI toxicities were 16% and 5%, respectively, in the RTOG 0126 trial (79.2 Gy arm). The incidence rates of late grade 2 and 3 GU toxicities were 0.5% and 0%, respectively, in the J-CROS trial, which were more favorable than photon therapy. In our study, no patient suffered from GI toxicity within the follow-up period, probably due to the short follow-up time. Ishikawa’s study showed that 81% of late toxicities occurred within 2 years after CIRT. The median follow-up time of patients in the low- and high-dose groups was 49 and 17 months, respectively (25). Therefore, toxicities were evaluated for a sufficient period in the low-dose group but not in the high-dose group.

This study had several limitations. First, the results were retrospectively analyzed from a single institution. A phase II study with a fixed dose of D_RBE_ = 65.5 Gy in 16 fractions is ongoing at our center. However, multicenter prospective studies are warranted to validate the safety and efficacy of carbon ion with the LEM model. Second, with 30.2 months follow-up, we could only assess acute and early late toxicities but not the long-term outcomes. Third, the sample size was small. Hence, the recruitment of more patients and a longer follow-up period are highly recommended.

## Conclusions

In conclusion, the short-term results of spot-scanning carbon ion therapy for localized prostate cancer were encouraging. Our results provide additional and useful information on the feasibility and safety of CIRT with LEM model for patients with localized prostate cancer. Longer follow-up periods and multicenter prospective studies are warranted to confirm the biochemical control and survival benefit of this promising technique.

## Data Availability Statement

The raw data supporting the conclusions of this article will be made available by the authors, without undue reservation.

## Ethics Statement

The studies involving human participants were reviewed and approved by Shanghai Proton and Heavy Ion Center Institutional Reviewer Board. The patients/participants provided their written informed consent to participate in this study.

## Author Contributions

PL, SF, and QZ conceived and designed the study. PL contributed data analysis and reagents. YL performed the physical data analysis. PL and ZH participated in patient treatment and follow-up. PL wrote the manuscript. SF and QZ reviewed the manuscript and contributed to the final version of the manuscript. All authors contributed to the article and approved the submitted version.

## Funding

Funding for this study was provided by the National Natural Science Foundation of China (81773225), Shanghai Municipal Health Commission (No. 201940121), and Pudong New Area Science and Technology Development Foundation (No. PKJ2020-Y52).

## Conflict of Interest

The authors declare that the research was conducted in the absence of any commercial or financial relationships that could be construed as a potential conflict of interest.

## Publisher’s Note

All claims expressed in this article are solely those of the authors and do not necessarily represent those of their affiliated organizations, or those of the publisher, the editors and the reviewers. Any product that may be evaluated in this article, or claim that may be made by its manufacturer, is not guaranteed or endorsed by the publisher.
